# An Approach towards Increasing Prediction Accuracy for the Recovery of Missing IoT Data based on the GRNN-SGTM Ensemble †

**DOI:** 10.3390/s20092625

**Published:** 2020-05-04

**Authors:** Roman Tkachenko, Ivan Izonin, Natalia Kryvinska, Ivanna Dronyuk, Khrystyna Zub

**Affiliations:** 1 Department of Publishing Information Technologies, Lviv Polytechnic National University, 12 Bandera str., 79000 Lviv, Ukraine; roman.o.tkachenko@lpnu.ua; 2 Department of Information Systems, Faculty of Management, Comenius University in Bratislava, 82005 Bratislava 25, Slovakia; 3 Department of e-Business, School of Business, Economics and Statistics, University of Vienna, A-1090 Vienna, Austria; Natalia.Kryvinska@fm.uniba.sk; 4 Department of Automated Control Systems, Lviv Polytechnic National University, 12 Bandera str., 79000 Lviv, Ukraine; ivanna.m.droniuk@lpnu.ua; 5 Center of Information Support, Lviv Polytechnic National University, 12 Bandera str., 79000 Lviv, Ukraine; khrystyna.v.zub@lpnu.ua

**Keywords:** IoT sensors, missing data, ANN techniques, data imputation, GRNN, Successive Geometric Transformation Model, non-iterative training, neural-like structures, hybrid systems, weighted summation

## Abstract

The purpose of this paper is to improve the accuracy of solving prediction tasks of the missing IoT data recovery. To achieve this, the authors have developed a new ensemble of neural network tools. It consists of two successive General Regression Neural Network (GRNN) networks and one neural-like structure of the Successive Geometric Transformation Model (SGTM). The principle of ensemble topology construction on two successively connected general regression neural networks, supplemented with an SGTM neural-like structure, is mathematically substantiated, which improves the accuracy of prediction results. The effectiveness of the method is based on the replacement of the summation of the results of the two GRNNs with a weighted summation, which improves the accuracy of the ensemble operation in general. A detailed algorithmic implementation of the ensemble method as well as a flowchart of its operation is presented. The parameters of the ensemble operation are determined by optimization using the brute-force method. Based on the developed ensemble method, the solution of the task of completing the partially missing values in the real monitoring dataset of the air environment collected by the IoT device is presented. By comparing the performance of the developed ensemble with the existing methods, the highest accuracy of its performance (by the parameters of Mean Absolute Percentage Error (MAPE) and Root Mean Squared Error (RMSE) accuracy) among the most similar in this class has been proved.

## 1. Introduction

The concept of communication of objects that use state-of-the-art technology to interact with one another and the environment [1] has drawn considerable attention both in the academic environment and in industry. Currently, the application of Internet of Things (IoT) technologies has been successfully implemented in such fields as manufacturing, trade, banking, medicine, infrastructure, etc.

From a technological perspective, an IoT is a network of connected devices that can interact. The modern state of the industrial internet makes it possible to integrate a number of devices with different encoders into one entity [2]. Thus, a peculiar network is formed above the object of attention that all the devices are focused on. Within this network, there is a constant collection, processing, and exchange of information, based on which decisions are automatically made on the management of the object.

One of the main problems of such systems is uncertainty, which can manifest itself also in the form of incomplete information about the object of attention. It arises for a variety of reasons.

Based on the conducted analysis of literature sources, in Table 1, it is summarized the main reasons for the incompleteness of datasets in the IoT systems.

As can be seen from Table 1, there are many reasons for the incompleteness in the datasets collected by IoT devices. The incompleteness of the datasets on the basis of which a specific decision is made on the management of the object of attention prevents the effective operation of such systems [17,18]. This leads to the wrong decisions made on the basis of such an analysis and makes it impossible to implement efficient processes of automation of different processes in automated control systems based on IoT [19,20]. Taking this into account, the problem of completing omissions in datasets is perhaps one of the most important preliminary processing procedures for the effective functioning of IoT systems.

## 2. Related Works

There are many different approaches to solving the task of completing missing values in datasets with the unanimous goal to produce a solution as accurately as possible [21,22]. In particular, in [3], the authors use their ST-correlated proximate model to solve the problem of completing missing data. The authors demonstrate a significant increase in the accuracy of completing missing data using their model compared to existing static methods (single imputation and multiple imputations).

In [23,24], the authors investigated the *k*-nearest neighbors method to complete missing data for different applications. This method allows one to replace the missing value in a dataset based on the *k* most similar to it. In [24], the effectiveness of applying this method for different values of *k* and different percentages of missing data in the set is investigated. Similar results were also obtained in [25]. Authors’ experimental studies performed on five different datasets to compare the effectiveness of using different methods of completing missing data have shown the best results for the *k*-nearest neighbors method. Moreover, the authors have shown the quality of its outcome to be not influenced by the dataset [25].

Nevertheless, in [26], one indicates that the *k*-nearest neighbors implicitly assumes that missing values are uniformly distributed at random in the dataset. However, this approach cannot be applied in the majority of cases. To improve this algorithm, in [26], a modification was developed that suggests significantly higher accuracy rates, as opposed to the basic algorithm. A fuzzy rule-based model was developed in [27] designed to complete missing data collected by IoT devices. The method shows much higher accuracy in comparison with the *k*-nearest neighbors method. The effectiveness of such models is confirmed in [28,29].

In [30], a method for rectifying omissions in the data collected by an IoT device using a new computational intelligence tool, the neural-like structures of the Successive Geometric Transformation Model (SGTM), is developed. The latter being universal approximators [31] are based on the principles of fast non-iterative learning performed in a predetermined number of steps and ensures the repetition of a solution. The authors have adopted a linear version of SGTM neural-like structures [30] to rectify omissions in the dataset as to the chemical composition of the air environment. The use of this computational intelligence tool has been shown to significantly increase the prediction accuracy in comparison with the arithmetic mean algorithm under satisfactory timing.

A modification of the above-mentioned method is presented in [32]. The authors have proposed to use the Kolmogorov–Gabor polynomial [33] as a tool for nonlinear input expansion. The SGTM neural-like linear structure was used as a fast means to find the coefficients of this polynomial. Considering the high approximation properties of the Kolmogorov–Gabor polynomial, the outcome of the method demonstrates higher accuracy, in comparison with the existing methods of machine learning, in particular. Additionally, higher polynomial degrees increase the accuracy of the method. However, due to the considerable expansion of the inputs of an SGTM neural-like structure (under high polynomial degrees), the ratio of the growth of time resources to the increase in the accuracy of the outcome of the method becomes unjustified.

In [34], a solution to the task of completing omissions in the data collected by an IoT device using a General Regression Neural Network (GRNN) is provided. This computational intelligence tool, with its high generalization properties, demonstrates the improved accuracy of previous methods. However, this method also suggests the need to use considerable memory resources for its operation. In [35], to reduce the memory cost of the GRNN model, the authors proposed to use an incremental learning method. The authors proposed an algorithm for dynamically adjusting global and local estimations and a polynomial extrapolation scheme for improving the quality of extreme value estimation. The last scheme is implemented in the hidden GRNN layer.

In [36], a new GRNN scheme with extended inputs is developed. The application of the Ito decomposition [33] in this case introduces a number of advantages over the existing input expansion schemes and provides high accuracy of the performance of this type of networks. However, the operating time of the method in general, as in the case of [32], depends on the number of members of this decomposition. In addition, the memory resources required for its operation are much higher than in [34] due to the significant space dimension increase of the input data. Therefore, the developed method requires accurate determination of the optimal parameters in each case separately.

In general, the approach to solving the task using GRNN is not new, with refinements and modifications of neural networks of this type [37,38,39,40,41,42,43] seeming promising, taking into consideration their advantages over neural networks of other types. These advantages can be represented as follows [36,43]:

- Lack of training procedures;
- The need to configure a single neural network parameter;
- Generalization properties are the highest among the known neural networks.
- Like any neural network, GRNN has a number of disadvantages including the following:
- Relatively low accuracy;
- Certain time delays in the application mode;
- No extrapolative properties.

Considering the velocity performance of modern computers, as well as the ability to apply cluster technologies to solve tasks using this type of neural network on separate clusters, the main desirable disadvantage of GRNN networks to be minimized is significant operating errors, which provides a basis for the research described in this paper.

Therefore, the purpose of this work is to improve the accuracy of completing omissions in the data collected by the Internet of Things device, which reduces the total prediction error.

The main contributions of this paper can be summarized as follows:

1. Based on the topology of two sequentially connected GRNN networks and an SGTM neural-like structure, a new ensemble method for solving prediction problem is devised; the introduction of the latter into the ensemble improves the accuracy of the prediction results by replacing the summation of the outcome of the two GRNNs with weighted summation with displacement;
2. The optimal operation parameters of the developed ensemble are selected by means of optimization, which provide the highest accuracy in solving the task;
3. The effectiveness of applying the developed ensemble is substantiated by a comparison between its outcomes and the latest existing developments dealing with solving the problem of completing the missing data in a real sample collected by an IoT device.

## 3. Materials and Methods

The General Regression Neural Network was introduced by Donald F. Spercht in 1991 [44]. This neural network can be used to model very irregular, substantially nonlinear response surfaces. Since its inception, it or its hybrids have been widely applied to solve various practical problems [41,42].

### 3.1. Fundamental Statements of GRNN

To analyze some basic features of the GRNN algorithm [34,37], let us consider a determined set of observations for a particular phenomenon or object. Each observation contains a vector of independent variables $\bar{x}$ and a dependent component −y. For the certain number of observations from a set, the values of the desired component are known, with others not containing separate values for the reasons described in Table 1. The task consists in predicting the values of the unknown dependent component for a particular observation:

(1) $$y=f(\bar{x}),$$

using a neural network.

If a set of observations is presented in matrix form $X={\left(\bar{x_{1}},\;\bar{x_{2}},\;\ldots \;,\;\bar{x_{N}}\right)}^{T}$, the production of response $y_{k}$ based on the relevant $\bar{x_{k}}$ taking into account the known part of the set $\bar{x_{i}}$ and $y_{i}$ can be performed using the GRNN method.

This involves the following steps [34]:

- Search for Euclidean distances from the input vector with components $x_{k,j}$ to available vectors with known output values $x_{i,j}$ that are considered to be support ones [34]:
  (2) $$E_{k,i}=\sqrt{\sum_{j=1}^{n}{\left(x_{k,j}-x_{i,j}\right)}^{2}}i,$$
  where $i=\bar{1,N}$ is a number of support vectors (observation) whose output values y are always known; $j=\bar{1,n}$ is a number of an input vector feature of each observation; $k=\bar{1,k_{\max}}$ is a number of an input vector (observation) whose output values y are unknown.
- Calculating Gaussian functions of Euclidean distances (2) [34]:
  (3) $$G_{k,i}=\exp\left(-\frac{{\left(E_{k,i}\right)}^{2}}{\sigma^{2}}\right),$$
  where σ is a smooth factor (*σ* > 0).
- Calculating the desired value $y_{k}$ according to a calculation formula of the GRNN method [34]:
  (4) $$y_{k}^{pred}=\frac{\sum_{i=1}^{N}y_{i}G_{k,i}}{\sum_{i=1}^{N}G_{k,i}}.$$

**Figure 1:**
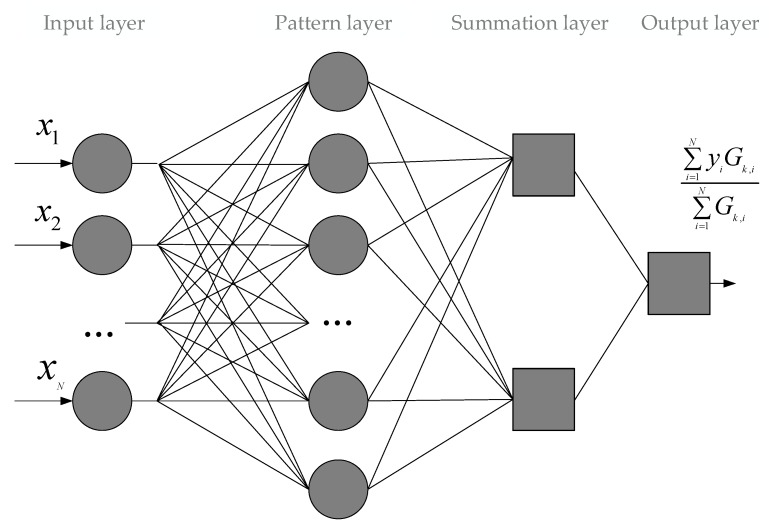
General Regression Neural Network (GRNN) topology.

The topology of this computational intelligence tool is shown in Figure 1.

### 3.2. Components of GRNN Output Generation Error

Let us analyze the component of the method error of the GRNN output signal generation. To implement this, the obvious identity is considered:

(5) $$\frac{\sum_{i=1}^{N}(y_{k}-y_{i})G_{k,i}}{\sum_{i=1}^{N}G_{k,i}}=y_{k}-\frac{\sum_{i=1}^{N}y_{i}G_{k,i}}{\sum_{i=1}^{N}G_{k,i}}.$$

Let us introduce the following notation:

(6) $$z_{k,i}=(y_{k}-y_{i})G_{k,i}.$$

Taking Notation (6) into account, Formula (5) can be represented as follows:

(7) $$y_{k}=\frac{\sum_{i=1}^{N}y_{i}G_{k,i}}{\sum_{i=1}^{N}G_{k,i}}+\frac{\sum_{i=1}^{N}z_{k,i}G_{k,i}}{\sum_{i=1}^{N}G_{k,i}}.$$

The first term of the right-hand side of Equation (7) corresponds to Formula (4) of the output signal calculation by the GRNN network. It is logical to assume the second term of the formula to reflect an error of the GRNN method provided in Equations (6) and (7) are accurate:

(8) $$\Delta_{k}=\frac{\sum_{i=1}^{N}z_{k,i}G_{k,i}}{\sum_{i=1}^{N}G_{k,i}}.$$

The known component of a method error, the difference between the exact value and the ones found by Formula (4), can also be calculated by Equation (8), but only for each of the *N* support vectors. However, this formula shows that the response surface of an error is sufficiently smooth [45,46] and, therefore, can be simulated somehow in the local region of the space of input variables. As experiments confirmed, the use of another GRNN network with a reduced value of a smooth factor σ provides a satisfactory approximation of the method error. Let us take into account that to improve the accuracy of the calculation of error value according to the formula:

(9) $$\Delta_{k}^{pred}\approx \frac{\sum_{i=1}^{N}(y_{i}^{pred}-y_{i})G_{k,i}}{\sum_{i=1}^{N}G_{k,i}}.$$

it is necessary to choose much smaller values of a smooth factor than when applied Formula (4), which is explained by the differences of reliefs of the response surfaces of the multivariate function and its method error.

### 3.3. GRNN Ensemble Using Two ANNs

The above-mentioned entails the method of increasing the accuracy of solving a regression problem based on a two-element GRNN ensemble using the general concept of applying networks of this type [43]. It consists of two main stages: data preparation and application procedures.

The procedure of preliminary data preparation involves the following steps:

To calculate the response according to the GRNN method for each i-th point of reference $i=\bar{1,N}$ by turns relative to the remaining *N*−1 points ($l=\bar{1,N-1}$):

(10) $$y_{i}^{pred}=\frac{\sum_{l=1}^{N-1}y_{l}G_{i,l}}{\sum_{l=1}^{N-1}G_{i,l}}.$$

To calculate values of deviation between exact and calculated values:

(11) $$\Delta_{i}=y_{i}-y_{i}^{pred}.$$

The procedure for applying two GRNNs to a current *k*-th vector requires the implementation of the following steps:

To calculate $y_{k}^{pred}$ by applying Equation (4);

To apply the following GRNN formula iteratively to predict an error:

(12) $$\Delta_{k}^{pred}=\frac{\sum_{i=1}^{N}\Delta_{i}G_{k,i}}{\sum_{i=1}^{N}G_{k,i}}.$$

A definitive outcome of the method is obtained according to the following formula:

(13) $$y_{k}\approx y_{k}^{pred}+\Delta_{k}^{pred}.$$

### 3.4. Linear SGTM Neural-Like Structure

The paper suggests the use of an additional linear correction neural-like structure based on the Successive Geometric Transformation Model in order to increase the accuracy of the task of completing omissions in the data collected by IoT devices.

Such an increase in the outcome accuracy of the method is possible due to replacing Summation (13) of the two predicted components by two GRNNs with a weighted summation with displacement (additional linear neural network):

(14) $$y_{k}=a_{0}+a_{1}y_{k}^{pred}+a_{2}\Delta_{k}^{pred},$$

where $a_{0},\;a_{1},\;a_{2}$ are coefficients of a weighted summation with displacement that are found by an SGTM neural-like structure.

**Figure 2:**
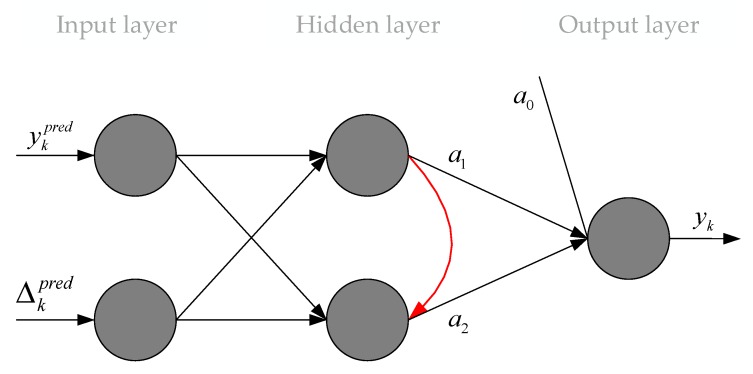
Topology of additional correction linear neural-like structure of the Successive Geometric Transformation Model (SGTM).

The topology of this computational intelligence tool is shown in Figure 2. Details of the greedy algorithm of training and functioning are given in [31].

It has been experimentally established [43] that a certain positive effect is achieved for the simplified variant of correction where a component summation according to Formula (13) is used. The modeling error using the GRNN network is affected by the inaccuracy of the network itself, with nonlinear deviations being somehow minimized by adequate parameter σ selection. On the other hand, the surface of error response approximated by the described method has turned out to contain systematic and linear components of deviations, which are largely eliminated by applying the SGTM neural-like structure of linear type (for a weighted summation with displacement) [31].

### 3.5. Proposed GRNN-SGTM Ensemble

**Figure 3:**
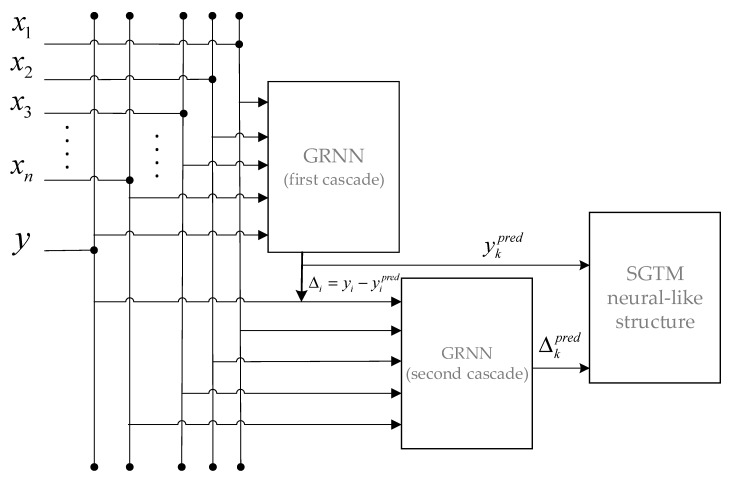
Flowchart of the GRNN–SGTM ensemble for solving the stated task.

On the basis of all the above, one proposes an ensemble of two GRNN networks and an SGTM neural-like structure. The flowchart of the ensemble operation is shown in Figure 3.

The application of the ensemble developed by authors will improve the accuracy of completed omissions in the data collected by IoT devices.

## 4. Modeling and Results

To do numerical calculations, a laptop with the Intel Core i5-600U processor (2.40 GHz), p 8.00 GB RAMM, and a 64-bit operating system was used.

### 4.1. Data Descriptions

Experimental studies on the performance of the developed ensemble have been conducted using a dataset collected by an IoT device [47]. Hourly chemical composition of the air was collected by chemical sensors of the IoT device in the area near the Italian city. Details of the data collection process are given in [47]. The attributes of this set and their main characteristics are given in Table 2 [34,36].

All the vectors with omissions have been removed. Thus, the simulation was implemented on the set of 6950 vectors [34]. The training and test samples were obtained by dividing the dataset randomly in the ratio of 80–20%. The first sample of data was used for training, the second sample for testing.

Given that the most missing values were in the CO column, the simulation was performed to recover the lost data of this attribute [34,36,43].

### 4.2. Performance Evaluation Indicators

To evaluate and analyze the outcomes of the developed method, the following indicators are used [34]:

1. Root Mean Squared Error (RMSE):
  (15) $$RMSE=\sqrt{\sum_{i=1}^{N}{\left(y_{i}^{p}-y_{i}\right)}^{2}},$$
2. Mean Absolute Percentage Error (MAPE):
  (16) $$MAPE=\frac{1}{N}\sum_{i=1}^{n}|\frac{y_{i}^{p}-y_{i}}{y_{i}}|100,$$
  where $y_{i}$ is an actual value and $y_{i}^{p}$ is an obtained value for each i vector.

### 4.3. Choice of Optimal Parameters of Ensemble

A General Regression Neural Network is characterized by the only setting parameter, namely the smooth factor σ. Accordingly, the proposed method based on an ensemble of two GRNNs will also depend on the value of this parameter. The SGTM neural-like structure operates in learning and application modes. Thus, the developed ensemble will also operate in both modes.

In this paper, optimization according to a brute-force method was performed to determine the smooth factor (σ) for the respective Gaussian functions of both GRNN networks. The SGTM neural-like structure (Figure 2) took the following parameters: the number of inputs is 2, the number of neurons in a hidden layer is 2, and one output. The number of inputs of both GRNNs in the ensemble is 11.

Let us denote by $\sigma_{1}$ the parameter of the smooth factor of the main GRNN in the ensemble, and by $\sigma_{2}$ the additional one. The experiment was done under changing values $\sigma_{1}\;(\sigma_{1}\in \left[0.01,1.49\right],\;\Delta_{\sigma_{1}}=0.01)$ and $\sigma_{2}\;(\sigma_{2}\in \left[0.01,1.49\right],\;\Delta_{\sigma_{2}}=0.01)$ to calculate the MAPE and RMSE of the developed ensemble. This choice is based on [36].

**Figure 4:**
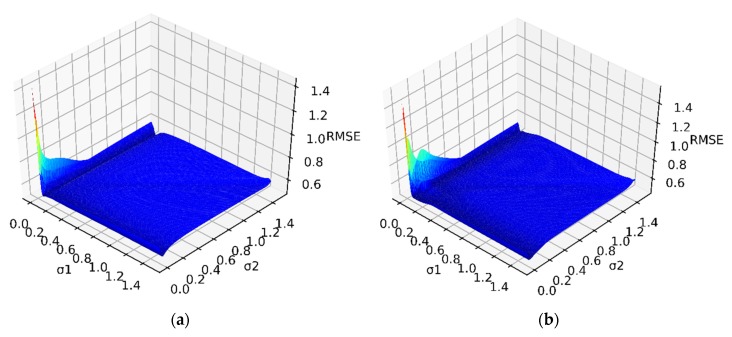
Root Mean Square Error (RMSE)-values under different combinations of smooth factors $\sigma_{1}$ та $\sigma_{2}$ of both GRNN ensemble networks: (**a**) in the training mode and (**b**) in the application mode.

**Figure 5:**
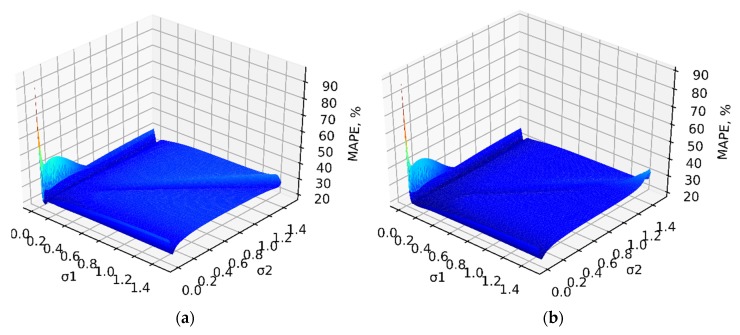
Mean Absolute Percentage Error (MAPE)-values under different combinations of smooth factors $\sigma_{1}$ та $\sigma_{2}$ of both GRNN ensemble networks: (**a**) in the training mode and (**b**) in the application mode.

The results obtained for both modes of ensemble operation based on indicators (15) and (16) are visualized in Figure 4 and Figure 5 respectively.

It should be noted that in Figure 4 and Figure 5 on the *ox* axis, different values of a smooth factor of the main GRNN network $\sigma_{1}$ are given. The *oy* axis represents the smooth factor of the additional GRNN network $\sigma_{2}$. The *oz* axis corresponds to the error values of RMSE (Figure 4) and MAPE (Figure 5) under different combinations of $\sigma_{1}$ and $\sigma_{2}$.

As can be seen from both surfaces (Figure 4 and Figure 5), there are local minima of the error surface. This can be traced in two cases:

Under the following parameters of ensemble operation:$\sigma_{1}$ is arbitrary, $\sigma_{2}\in [0.03,\;0.07]$;

Under the following parameters of ensemble operation: $\sigma_{2}$ is arbitrary, $\sigma_{1}\in [0.04,\;0.07]$.

The most accurate results were obtained in the first case. The optimal parameters of the proposed ensemble, as well as the respective values of indicators (15) and (16), under the modes of training and application, are given in Table 3.

These very results were taken into account while comparing the developed ensemble with the outcomes of existing methods.

## 5. Comparison and Discussion

The accuracy of the developed ensemble operation was compared with the outcomes of the state-of-the-art developments in the field of computational intelligence dealing with the problem of recovering missing data collected by Internet of Things devices. The most similar methods were selected, namely GRNN and SGTM neural-like structures, as well as modifications thereof. Detailed outcomes of the existing machine learning methods (SVM, AdaBoost, Random Forest, etc.) can be found in [32]. With them exhibiting significantly lower performance accuracies, they were not considered in this study.

The results of the comparison based on indicators (15) and (16) and by choice of the optimal parameters of each method are summarized in Table 4.

As can be seen from Table 4, the method of [30] demonstrates the least accuracy. However, its modification from [32] suggests a much smaller RMSE value. The method of completing missing data collected by the Internet of Things device based on GRNN [34], as well as the method based on its modification [36], shows approximately the same accuracy results based on MAPE, with the latter revealing a significantly higher RMSE value.

The best performance in terms of accuracy based on both indicators is demonstrated by the developed ensemble. The construction of two successive GRNNs, as well as the weighted summation of the results using the SGTM neural-like structure, made it possible to improve the operation accuracy of the solution of the problem of completing omissions in the data collected by the Internet of Things devices.

Moreover, given that GRNN is a neural network without training and SGTM neural-like structure training is non-iterative, i.e., high-speed, efficient hardware implementation of the ensemble for Artificial Intelligence of Things(AIoT)-based device construction is possible [48,49]. This will allow routine preliminary processing of the data inside the device, which will increase the performance of IoT systems in general.

## 6. Conclusions

A new computational intelligence tool has been developed to improve the accuracy of solving the task of completing omissions in the data collected by Internet of Things devices. It is based on the use of two General Regression Neural Networks and one SGTM neural-like structure. The purpose of the latter is to provide additional compensation for the constant displacement and linear component of the error of the response surface approximation formed by two successive networks by using an additional SGTM neural-like linear structure at the output of the ensemble.

The basic statements of the procedures of the GRNN network operation are described. The components of its output signal generation error have been analyzed. The application of the SGTM neural-like structure for a weighted summation of the outcome of the ensemble has been substantiated, which constitutes a basis for the detailed algorithmic implementation of the ensemble and the flowchart of its operation presented.

The outcome of the developed ensemble was tested on the actual data collected by the IoT device. The paper suggests a solution to the task of completing missing values in datasets of the monitoring composition of the air environment. Experimentally, the effectiveness of the developed ensemble in solving this task was established. Moreover, a comparison between the performance of the developed method and the performance of a number of existing ones was drawn. The highest precision of the developed method was established on the basis of both MAPE and RMSE.

There will be further studies conducted into the choice and testing of optimization methods that are more effective in terms of the timing characteristics for the choice of optimal parameters of developed ensemble operation. Besides, one should consider the possibility to design an AIoT-based hardware variant of the developed ensemble with a view to improving the operational efficiency of IoT-based systems, e.g., smart home, smart business, smart city, etc. This is possible due to transferring some basic preliminary processing operations by a device itself. In this case, the purpose and therefore the main function of the device will be changed from data collection to knowledge aggregation. This will significantly reduce loading on cloud services of data processing, which, in turn, will increase the performance of all subsystems based on them.

## Figures and Tables

**Table 1:** Reasons for the omission in data collected by IoT devices.

Reasons	Investigations
the unstable network communication, synchronization problems, unreliable sensor devices, environmental factors, and other device malfunctions;	[3,4,5,6]
the interruption of the data acquisition in long-term monitoring scenarios;	[7]
the location, firmware may not be consistent across locations. This could mean differences in reporting frequency or formatting of values;	[8]
the sensor failures, monitoring system failures or network failures;	[9]
the storage errors, unreliable IoT devices, unstable network status;	[10]
the incorrect response or nonresponse of the IoT-based sensors;	[11]
the collision of the nodes when the information passes from sender to receiver;	[12]
the channel effects and mobility of the end-devices;	[13]
the errors in data collection and transmission;	[14]
the data integration from different sources into a unified schema;	[15]
the lack of battery power, communication errors, and malfunctioning devices.	[16]

**Table 2:** The main characteristics of the Internet of Things (IoT)-based dataset.

Variable	MEAN Value	MAX Value	MIN Value	Chemical Nomenclature
Tungsten monoxide	817.0748	2683	322	WO
Tungsten dioxide	1452.494	2775	551	WO_2_
Titanium	958.2302	2214	390	Ti
Temperature	17.75942	44.6	0.1	T
Relative humidity	48.90163	88.7	9.2	RH
Non-methane hydrocarbons	1119.626	2040	647	SnO_2_
Nitrogen monoxide	250.465	1479	2	NO
Nitrogen dioxide	113.7894	333	2	NO_2_
Indium oxide	1057.363	2523	221	InO
Carbon monoxide	2.19059	11.9	0.1	CO
Benzene	10.54635	63.7	0.2	C_6_H_6_
Absolute humidity	0.986315	2.2345	0.1847	AH

**Table 3:** Optimal parameter of proposed ensemble operation.

$\sigma_{1}$	$\sigma_{2}$	MAPE, %	RMSE
0.23	0.05	20.268 (train mode)	0.493 (train mode)
		18.828 (test mode)	0.458 (test mode)

**Table 4:** Comparison of operation accuracy of all the methods investigated.

Method	Parameters	RMSE	MAPE, %
GRNN [34]	input neurons = 11, σ=0.06.	0.464	19.856
Extended-inputs GRNN [36]	input neurons = 78, σ=0.09.	0.549	19.905
SGTM neural-like structure (test mode) [30]	input neurons = 11, hidden neurons = 11 (1 hidden layer).	0.497	20.491
Extended-input SGTM neural-like structure (test mode) [32]	input neurons = 78, hidden neurons = 40 (1 hidden layer).	0.458	19.911
GRNN-SGTM ensemble (test mode)	parameters are given above in the text	0.458	18.828
